# Amphotropic murine leukemia virus is preferentially attached to cholesterol-rich microdomains after binding to mouse fibroblasts

**DOI:** 10.1186/1743-422X-3-21

**Published:** 2006-04-02

**Authors:** Christiane Beer, Lene Pedersen

**Affiliations:** 1 Institute of Clinical Medicine and Department of Molecular Biology, University of Aarhus, Aarhus, Denmark

## Abstract

**Background:**

We have recently shown that amphotropic murine leukemia virus (A-MLV) can enter the mouse fibroblast cell line NIH3T3 via caveola-dependent endocytosis. But due to the size and omega-like shape of caveolae it is possible that A-MLV initially binds cells outside of caveolae. Rafts have been suggested to be pre-caveolae and we here investigate whether A-MLV initially binds to its receptor Pit2, a sodium-dependent phosphate transporter, in rafts or caveolae or outside these cholesterol-rich microdomains.

**Results:**

Here, we show that a high amount of cell-bound A-MLV was attached to large rafts of NIH3T3 at the time of investigation. These large rafts were not enriched in caveolin-1, a major structural component of caveolae. In addition, they are rather of natural occurrence in NIH3T3 cells than a result of patching of smaller rafts by A-MLV. Thus cells incubated in parallel with vesicular stomatitis virus glycoprotein (VSV-G) pseudotyped MLV particles showed the same pattern of large rafts as cells incubated with A-MLV, but VSV-G pseudotyped MLV particles did not show any preference to attach to these large microdomains.

**Conclusion:**

The high concentration of A-MLV particles bound to large rafts of NIH3T3 cells suggests a role of these microdomains in early A-MLV binding events.

## Background

Retroviral vectors carrying the envelope protein of amphotropic murine leukemia virus (A-MLV) are some of the most widely used retroviral vector pseudotypes in gene therapy trials. Achievement of controlled but efficient gene delivery will, however, depend on a detailed insight into virus biology. We have previously shown that A-MLV entry is closely associated with cholesterol-rich microdomains like rafts and caveolae [1] and that A-MLV envelope protein is associated with rafts in infected cells suggesting a possible role of rafts in A-MLV assembly [2]. It has also been shown for other viruses that rafts and/or caveolae are important for their entry and assembly [3-8]; specifically, has caveola-mediated entry been shown for, e.g., SV40 [4], echovirus 1 [7], and human coronavirus 229E [8]. Both domains consist of high concentrations of cholesterol, sphingomyelin, ganglioside GM1, and other saturated lipids [9,10] but in contrast to rafts do caveolae build omega-shaped invaginations within the plasma membrane of cells [11]. The unique lipid composition of rafts and caveolae leads to the specific incorporation or exclusion of proteins in these domains thereby creating distinct microenvironments for cellular processes [10,11].

Studying SV40 entry it was found that viral entry via caveolae occurs through an endocytic mechanism and that it – in comparison to an endocytic entry via clathrin-coated pits – is a cholesterol-dependent, pH-independent, and slow process [4]. We also found these hallmarks of caveolae-mediated entry when studying A-MLV entry of fibroblastic cells [1]. Association of the viral receptor with caveolae would seem essential for viral entry through caveolae and our previous investigations also showed that the A-MLV receptor protein Pit2, a sodium-dependent phosphate transporter, is able to directly associate with caveolin-1 (cav-1) [1], one of the major structural proteins of caveolae [11]. However, the omega-like shape of caveolae and their average size of around 70 nm would suggest that A-MLV with its diameter of about 110 nm binds outside of caveolae. As rafts are suggested to be pre-caveolae [11] and a large fraction of the A-MLV receptor Pit2 was found associated with cholesterol-rich microdomains [1], we have here investigated if rafts and caveolae are involved in the early steps of A-MLV binding.

## Results

**Figure 1:**
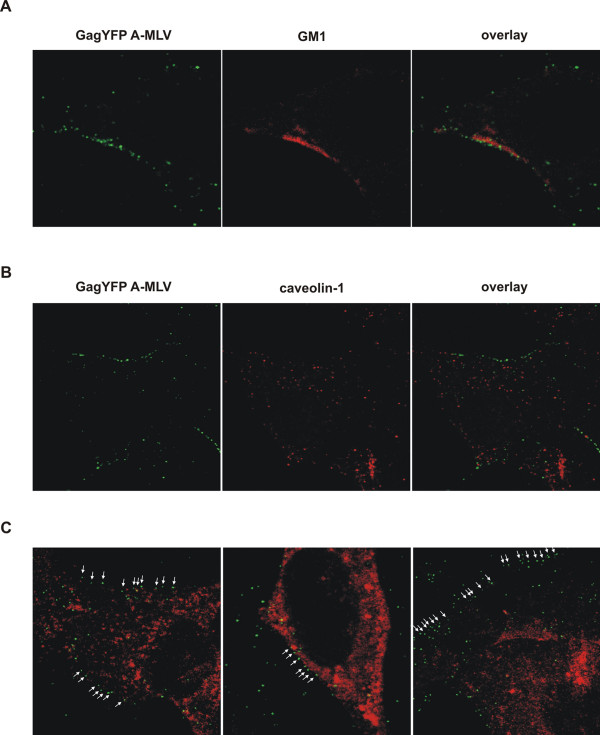
**A-MLV binds preferentially to large rafts**. **A) **NIH3T3 cells were incubated with GagYFP A-MLV (green) for 3 hours, fixed, and GM1 was stained with fluorescently labeled CTX (red). **B) **NIH3T3 cells were incubated with GagYFP A-MLV (green) for 3 hours and fixed. The cells were permeabilized with Triton X-100 and cav-1 was stained (red). **C) **NIH3T3 cells stably expressing cav-1 mRed fusion protein (red) were incubated with GagYFP A-MLV (green) for 3 hours and fixed. Clusters of viral particles as those found bound to large rafts in A are labelled with arrows. All pictures were taken using confocal microscopy.

First, we wanted to investigate if A-MLV binds to cholesterol-rich microdomains. Therefore, NIH3T3 cells were incubated for 3 hours at 37°C with fluorescently labeled A-MLV (GagYFP A-MLV) containing a nucleocapsid protein fused with yellow fluorescence protein (YFP) [12]. After subsequent washing and fixation, the cells were incubated with fluorescently labeled cholera toxin (CTX). This is a standard procedure for staining of cholesterol-rich microdomains since CTX binds specifically to GM1, a marker of rafts and caveolae [13]. As shown in figure 1A, cell-bound A-MLV showed a pronounced attachment to large GM1-positive microdomains. As GM1 is a general marker for cholesterol-rich microdomains, we investigated if these regions of preferred A-MLV binding were also enriched in caveolin-1 (cav-1), a major structural protein of caveolae. NIH3T3 cells were incubated with GagYFP A-MLV particles, washed, fixed, and permeabilized. Subsequently, the cells were stained for cav-1 and investigated using confocal microscopy. As expected a part of GagYFP A-MLV particles co-localized with cav-1 could be observed, however, cav-1 was not enriched at the favored binding sites of GagYFP A-MLV (Fig. 1B). The same was true for GagYFP A-MLV bound to NIH3T3 cells stably expressing a cav-1 mRed fusion protein (Fig. 1C). From these data, we suggest that rafts rather than caveolae are involved in the early steps of A-MLV binding.

**Figure 2:**
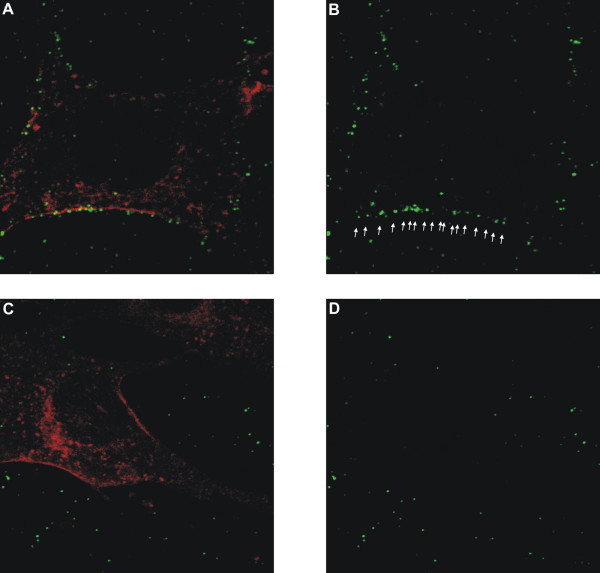
**Large rafts are present in NIH3T3 cells independent of A-MLV binding**. **A)**, and **B) **NIH3T3 cells were incubated with GagYFP A-MLV (green) for 30 min, fixed, and stained for GM1 with fluorescently labeled CTX (red). Clusters of viral particles bound to large rafts are labelled with arrows in B). **C)**, and **D) **NIH3T3 were incubated with VSV (green) for 30 min, fixed, and stained for GM1 with fluorescently labeled CTX (red). All images were taken using confocal microscopy. A) and C) are merged images, B) and D) show only GagYFP A-MLV or GagYFP VSV particles from A) and C), respectively.

Interestingly, in many investigated cells the stained cholesterol-rich microdomains appeared as large patched regions within the plasma membrane of the cells (Fig. 1A). As the cells were fixed before staining with CTX, we could exclude a patching of smaller rafts due to CTX binding. However, it is known, that binding of ligands or viruses to their raft-associated receptor can lead to patching of smaller raft domains [14,15]. We therefore investigated whether virus binding lead to patching of GM1-rich microdomains. As a control, we included VSV-G pseudotyped GagYFP MLV particles (here referred to as GagYFP VSV); VSV enters cells via clathrin-coated pits [16] and binding of these viral particles to the cells should therefore not lead to patching of cholesterol-rich microdomains. Thus, VSV or A-MLV pseudotypes of GagYFP MLV cores were added to NIH3T3 at 37°C, and after 30 minutes the cells were washed, fixed, and stained for GM1 with fluorescently labeled CTX. Confocal microscopy revealed that large cholesterol-rich microdomains were present in cells incubated with both VSV and A-MLV (Fig. 2). The same was true for NIH3T3 cells incubated with viral like particles lacking viral envelope proteins (data not shown). But in comparison to A-MLV, neither VSV nor viral like particles lacking viral envelope proteins showed preferential attachment to large rafts. Thus, while binding of A-MLV to the large raft regions seems to be A-MLV envelope specific, it did not lead to the formation of the large rafts.

**Figure 3:**
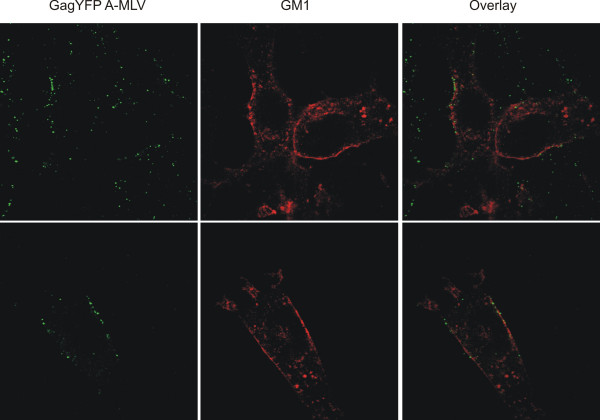
**A-MLV binding to large rafts is independent of extraction of plasma membrane cholesterol**. NIH3T3 cells were treated with 10 mM MBCD, washed, and incubated with GagYFP A-MLV (green) for 30 min. Subsequently, the cells were washed, fixed, and stained for GM1 using fluorescently labeled CTX (red). Images were taken using confocal microscopy.

As rafts are enriched in cholesterol and cholesterol has been shown to be important for A-MLV entry [1], we wanted to investigate, if cholesterol was important for the preferential binding of A-MLV to the large raft regions. Therefore, we treated NIH3T3 cells with 10 mM methyl-beta-cyclodextrin (MBCD), which is known to extract cholesterol out of the plasma membrane of eukaryotic cells [17]. After this treatment, the cells were incubated with GagYFP A-MLV for 30 min at 37°C, washed, fixed, and stained for GM1 with fluorescently labeled CTX. Although we have previously shown that this treatment is sufficient to extract up to 70 percent of the plasma membrane cholesterol of NIH3T3 cells [1], large raft regions were still present (Fig. 3). In addition, A-MLV showed the same binding pattern as in the experiments in figures 1 and 2 demonstrating that depletion of cholesterol alone was not sufficient to prevent A-MLV binding to the large raft regions.

## Discussion

The A-MLV replication cycle is closely associated with cholesterol-rich microdomains like rafts and caveolae. We have previously shown that A-MLV can enter mouse fibroblasts through caveola-mediated endocytosis and that the A-MLV envelope protein is associated with rafts in infected cells [1,2]. Here, we have furthermore demonstrated that rafts are involved in A-MLV binding.

Using confocal microscopy we found that A-MLV binds preferentially to large GM1-positive membrane regions of NIH3T3 cells, which are most likely rafts. Cav-1 staining of NIH3T3 cells as well as NIH3T3 cells stably expressing a cav-1 mRed fusion protein revealed that cav-1 was not enriched at the favored binding sites of A-MLV. Interestingly, the large rafts were not a result of virus induced aggregation of smaller rafts (raft patching). NIH3T3 cells incubated together with VSV or with viral particles lacking a viral envelope protein had the same large GM1-positive domains as cells incubated with A-MLV. As VSV enters and infects cells via clathrin-coated pits [16], binding of VSV should not lead to any patching of rafts. Therefore, we conclude that the presence of large rafts in NIH3T3 cells is not a result of A-MLV binding. Raft patching is especially known from investigations of the T cell receptor (TCR) and the T cell coreceptor CD4. It has been shown that crosslinking of TCR and CD4 by antibodies as well as incubation of T cells with CTX lead to raft patching [18-20]. As we fixed the cells prior to staining with CTX, we can exclude that the large rafts are artifacts caused by the staining procedure. It should also be noted that large rafts were not present in other cell lines like 293T (human kidney cell line) or MDTF (*Mus dunni *tail fibroblasts) regardless of A-MLV binding (data not shown).

The origin of the large rafts in NIH3T3 cells is not known. However, raft patching occurs mainly through ligand-receptor interactions or other protein-protein interactions [14,15] and it is possible that proteins from serum or the presence of a prominent extracellular matrix associated with rafts could lead to raft patching and the appearance of large rafts in NIH3T3 cells. Raft patching in NIH3T3 cells induced by pronounced protein-protein interactions would indeed explain the resistance of these large domains to extraction of plasma membrane cholesterol with MBCD. Thus, it is likely that a complex and rigid protein network could preserve large rafts from disintegration even in the absence of cholesterol. In addition, A-MLV binding to large rafts was independent of plasma membrane cholesterol indicating that the A-MLV receptor Pit2 or other virus interacting proteins were still present in these regions. All vector particles used in the present study were produced by transient transfection of 293T cells and since A-MLV but not VSV or viral particles lacking viral envelope proteins showed favored binding to large rafts, we conclude that this is due to the presence of A-MLV envelope protein on the particles and therefore most likely due to the presence of Pit2 in these regions. Furthermore, in comparison to A-MLV only a small amount of VSV particles were cell-bound at the time of investigation. This is probably due to the faster entry of VSV into cells since we have previously shown that VSV particles enter NIH3T3 cells 4 times faster than A-MLV [1]. In addition, to ensure that the reason for the observed differences in A-MLV and VSV binding was not due to a lesser amount of VSV particles we also have incubated cells with lesser quantities of A-MLV. As expected, the amount of cell-bound A-MLV decreased but a high amount of the cell-bound particles were still found attached to large rafts (data not shown).

While we previously demonstrated that the major entry route of A-MLV in NIH3T3 cells is via caveola [1], we here found that the vast majority of cell-bound A-MLV virions associated with GM1-positive regions and not with cav-1-positive regions. Indeed, the amount of A-MLV particles associated with large rafts within 30 minutes of virus exposure suggests that rafts are involved in early events of A-MLV binding and that A-MLV virions bind to the cells outside of caveolae and subsequently associate with caveolae in the entry process. Furthermore, in agreement with the slow infection kinetic of A-MLV these data also suggest that transport of A-MLV from rafts to caveolae is a limiting step in A-MLV entry.

## Conclusion

Taken together, our results show that A-MLV binds preferentially to large rafts in NIH3T3 suggesting involvement of these microdomains in early steps of A-MLV binding.

## Methods

### Cells

NIH3T3 cells (ATCC CRL-1658) were propagated in Dulbecco's modified Eagle's medium (DMEM) supplemented with glutamine and 10% Newborn Calf Serum (NCS). 293T cells (ATCC CRL-11268) were propagated in DMEM supplemented with glutamine and 10% Fetal Calf Serum (FCS). All cells were grown at 37°C, 10% CO_2 _and 95% humidity.

To stably express a cav-1 mRed fusion protein NIH3T3 cells were co-transfected with a construct encoding the cav-1 mRed fusion protein [21] and the pSV2pac plasmid encoding puromycin resistance gene [22]. After 14 days of selection with medium containing 2 μg/ml puromycin, the cells were propagated in normal cultivation medium and used for investigations.

### Virus production of GagYFP A-MLV and VSV

For production of Gag-YFP A-MLV or GagYFP VSV particles, 293T cells were seeded in T75 flasks and grown to 70% confluence. The cells were transiently co-transfected with a Gag-YFP construct, containing Moloney MLV Gag-gene encoding viral structural proteins and an YFP-tagged nucleocapsid protein [12], pHIT111 (β-galactosidase encoding retoviral vector) [23], and a pHIT derived vector encoding the A-MLV (4070A isolate) envelope or a plasmid encoding the VSV glycoprotein (Clontech). Forty-eight hours after transfection, the supernatants were filtrated (0.45 μm) and stored until use at -80°C.

### MBCD treatment

To extract cholesterol out of the plasma membrane, NIH3T3 cells were overlaid with 10 mM MBCD (Sigma). After 30 min at 37°C, the cells were washed once with cell culture medium and used in virus binding studies.

### Immunofluorescent staining

NIH3T3 cells were seeded on Chamber Slides (Nunc) and grown to 80% confluence. The cells were incubated with GagYFP A-MLV or GagYFP VSV for 0.5 hour or 3 hours as indicated. In some experiments, the cells were treated with MBCD before incubation with A-MLV. After binding of the viruses, the cells were washed with PBS, immediately overlaid with 4% paraformaldehyde, and incubated for 15 min at RT. The fixed cells were washed with PBS, blocked with PBS containing 10% horse serum and 3% bovine serum albumin, and incubated with an antibody against cav-1 (BD Bioscience and Transduction Laboratories). Subsequently, the cells were overlaid with Alexa Fluor 594 labelled secondary antibody (Molecular Probes), washed in PBS, and the slides were mounted with immunofluorescence mounting medium (Dako).

For staining of GM1, the cells were blocked with PBS containing 10% horse serum and 3% bovine serum albumin and incubated with Alexa Fluor 594-conjugated cholera toxin (4 μg/ml) (Molecular Probes).

The confocal images were captured with a Leica TCS SP confocal Microscope (Leitz). YFP and Rhodamine were excited individually using argon laser 488 nm line and green helium neon laser 543 nm line, respectively. The two single-color images were subsequently merged into an RGB-image. Brightness and contrast were adjusted.

## Competing interests

The author(s) declare that they have no competing interests.

## Authors' contributions

Both authors conceived of the study and drafted the manuscript. CB carried out the experimental work.
